# Seasonal Variation in the Assembly Mechanisms and Co-Occurrence Patterns of Bacterial Communities in Water and Sediments of Reservoirs in Arid and Semi-Arid Regions

**DOI:** 10.3390/microorganisms14081657

**Published:** 2026-07-29

**Authors:** Junlin Dong, Xiaocong Qiu, Juan Yin, Cheng Xu, Qiangqiang Yang, Kai Wang, Zengfeng Zhao

**Affiliations:** 1 School of Civil and Hydraulic Engineering, Ningxia University, Yinchuan 750021, China; 12024131357@stu.nxu.edu.cn (J.D.);; 2 School of Life Science, Ningxia University, Yinchuan 750021, China

**Keywords:** bacterial communities, arid and semi-arid reservoirs, water–sediment habitats, community assembly, co-occurrence networks

## Abstract

Reservoir water and sediments are interconnected but ecologically distinct microbial habitats. Their seasonal bacterial dynamics and assembly processes remain poorly understood in arid and semi-arid regions. We investigated bacterial communities in the water and surface sediments of eight reservoirs in Guyuan, Ningxia, in April, July, and October 2025 using 16S rRNA gene sequencing. Water and sediment communities differed markedly in composition. Sediments had substantially higher α-diversity than water: mean Chao1 and Shannon indices were 3063.12 and 9.98 in sediments, compared with 748.38 and 6.25 in water. Seasonal turnover was stronger in water. Water communities differed significantly between April and July (R^2^ = 0.1139, *p* = 0.001) and between April and October (R^2^ = 0.0968, *p* = 0.013), whereas sediment communities did not differ between April and July (R^2^ = 0.0581, *p* = 0.877). Water communities were also more closely associated with seasonal changes in temperature, pH, and nutrients. βNTI-based null models indicated that water communities were mainly associated with stochastic processes sensu lato, whereas sediments showed a stronger deterministic signal, particularly heterogeneous selection. Sediment co-occurrence networks were more complex; in July, they contained 10,496 edges, compared with 7005 edges in water. Network complexity peaked in July in both habitats. These findings show that sediments act as relatively diverse and stable microbial reservoirs, whereas water communities respond more strongly to seasonal environmental variation. They clarify how habitat and season jointly structure bacterial communities in arid and semi-arid reservoirs.

## 1. Introduction

Reservoirs are important semi-artificial aquatic ecosystems that provide flood control, irrigation, hydropower generation, and domestic water supplies [1,2,3]. However, dam construction and reservoir operation can alter hydrological connectivity, water residence time, sediment transport, nutrient retention, and physicochemical conditions [4,5]. These changes may increase environmental heterogeneity [6] and affect biogeochemical processes in both the water column and sediments [7]. Such effects are particularly important in arid and semi-arid regions, where limited precipitation, strong evaporation, and weak inflow can further modify reservoir hydrodynamics and nutrient cycling [8].

Microorganisms play central roles in organic matter transformation, nutrient cycling, and ecosystem functioning in freshwater environments [9,10]. Bacterial community composition is shaped by both environmental conditions and ecological assembly processes. Deterministic processes [11], such as environmental filtering, may select taxa adapted to specific habitats, whereas stochastic processes, including dispersal and ecological drift [12], may generate community variation when selection is weak [13]. Understanding how these processes vary between water and sediment habitats is therefore essential for interpreting microbial community dynamics in reservoir ecosystems.

Despite growing interest in freshwater microbial ecology, several knowledge gaps remain. First, many studies of reservoir microorganisms have focused on either water or sediments alone, whereas relatively studies have compared paired water–sediment habitats within the same reservoir system [14,15]. Second, most available studies have been conducted in humid, subtropical, or otherwise intensively studied regions [16], whereas reservoirs in arid and semi-arid areas remain underrepresented [17]. Third, the seasonal responses of microbial community composition, assembly processes, and co-occurrence patterns in water-scarce reservoir ecosystems are still poorly understood. These gaps limit our understanding of how habitat heterogeneity and seasonal hydrological variability jointly structure reservoir bacterial communities. Based on these knowledge gaps, arid and semi-arid reservoirs provide a useful system for examining how habitat type and seasonal hydrological variability jointly shape bacterial communities. Unlike humid or temperate reservoirs, these systems are characterised by limited precipitation, strong evaporation, weak inflow, and concentrated summer rainfall [18]. We therefore hypothesised that sediments would support higher bacterial diversity, more complex co-occurrence network structures, and a stronger deterministic assembly signal because of their greater environmental stability, nutrient accumulation, and microhabitat heterogeneity. In contrast, water communities were expected to exhibit stronger seasonal turnover and a greater contribution of stochastic processes sensu lato because the water column is more directly exposed to short-term hydrological and environmental changes.

The Ningxia Hui Autonomous Region is located in the arid and semi-arid inland region of northwest China. In southern Ningxia, reservoirs are important sources of irrigation and domestic water, but they are also strongly influenced by seasonal precipitation, evaporation, and hydrological regulation [8].

In this study, we investigated paired bacterial communities in the water and surface sediment bacterial communities from eight reservoirs in Guyuan, Ningxia, during April, July, and October 2025. We tested these expectations by integrating analyses of community composition analysis, environmental correlation analysis, beta nearest taxon index (βNTI) null-model analysis, and co-occurrence network analysis. This study provides new insight into how habitat differentiation and seasonal environmental variability jointly structure bacterial communities in arid and semi-arid reservoir ecosystems.

## 2. Materials and Methods

### 2.1. Study Area

**Figure 1:**
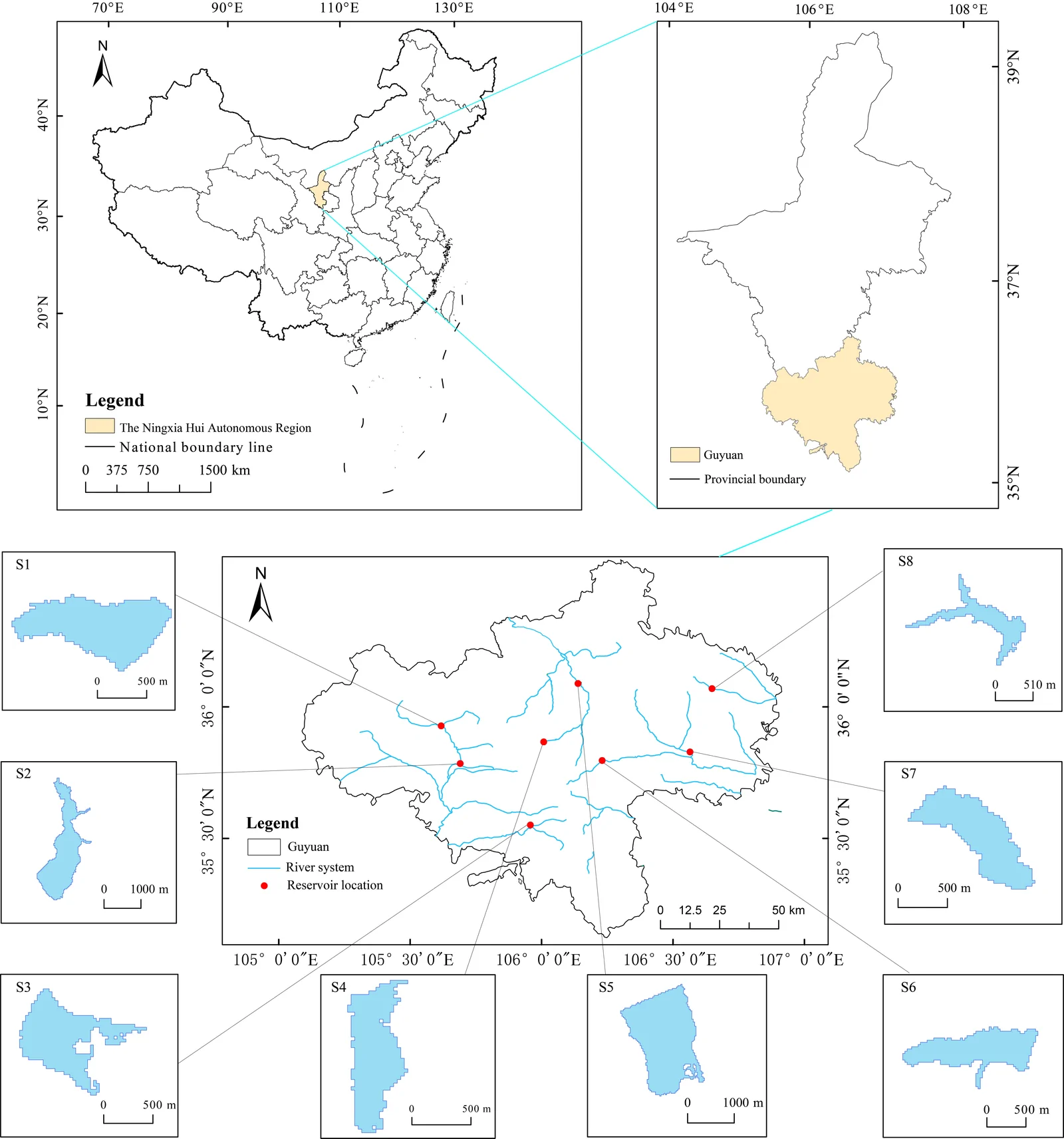
Layout of Sampling Points in the Reservoir.

Guyuan City, in the Ningxia Hui Autonomous Region, is located between 105°19′–106°57′ E and 35°14′–36°31′ N and has a temperate, dry, semi-arid climate. Mean annual precipitation is approximately 400–500 mm; however, evaporation greatly exceeds precipitation, and groundwater resources are limited, resulting in severe water scarcity. Reservoirs therefore serve as essential sources of irrigation water. Eight representative reservoirs were selected on the basis of storage capacity, functional importance, and spatial coverage (Figure 1). One fixed sampling station was established in each reservoir to minimise the influence of obvious direct anthropogenic point-source inputs. Detailed location information for each reservoir is provided in Table S1.

### 2.2. Sample Collection

Water and sediment samples were collected from each reservoir in April, July, and October 2025. All three sampling campaigns were conducted at the same fixed stations in the eight reservoirs to ensure spatial consistency among seasons. During the study period, the reservoirs were under similar routine operating conditions, and no unusual events—such as extreme precipitation, emergency reservoir-management interventions, visible pollutant discharges, or other abnormal disturbances—were recorded or observed at the sampling sites.

At each sampling location, three water subsamples were collected 0.5 m below the surface using a water sampler and thoroughly mixed to obtain one composite water sample. The composite sample was then divided into two portions. One portion was vacuum-filtered through sterile 0.22 μm membranes, rapidly frozen in liquid nitrogen or on dry ice, and stored at −80 °C for DNA extraction and high-throughput sequencing. The other portion was refrigerated for physicochemical analysis. Sediment samples were collected using a modified Peterson grab sampler. Three randomly collected surface-sediment subsamples from the upper 10 cm were thoroughly mixed to obtain one composite sediment sample. The sediment samples were transported to the laboratory, freeze-dried, ground, passed through a 100-mesh sieve, and stored at 4 °C until subsequent analysis.

The pooled sub-samples were used to reduce small-scale spatial heterogeneity within each sampling site and were not treated as independent statistical replicates. Thus, the statistical unit was defined as one composite sample from each reservoir in each season and habitat, resulting in a total of 48 composite samples, corresponding to 8 reservoirs × 3 seasons × 2 habitats.

### 2.3. Physical and Chemical Parameter Analysis

Water pH, water temperature (WT), electrical conductivity (EC), and salinity (SAL) were measured in situ using a YSI Pro Plus portable water-quality analyser (Shanghai, China), whereas fluoride (F^−^) concentrations were measured on site using a HACH HQ40d portable water-quality analyser. Additional water-quality parameters were determined in accordance with the relevant Chinese national or industry standards: total nitrogen (TN; HJ 636-2012), total phosphorus (TP; GB/T 11893-1989), ammonia nitrogen (NH_3_-N; HJ 535-2009), nitrate nitrogen (NO_3_^−^-N; HJ 198-2024), and the permanganate index (COD_Mn_; GB 11892-1989).

pH (HJ 962-2018), electrical conductivity (EC; HJ 802-2016), fluoride (F^−^; HJ 873-2017), total nitrogen (TN; HJ 717-2014), total phosphorus (TP; HJ 632-2011), ammonium nitrogen (NH_4_^+^-N; NY/T 1848-2010), and nitrate nitrogen (NO_3_^−^-N; HJ 634-2012) were quantified in sediment samples in accordance with the relevant standards.

Quality assurance and quality control procedures were implemented throughout the physicochemical analyses. For in situ measurements, including EC and SAL, portable instruments were calibrated before use according to the manufacturers’ instructions and the relevant standard procedures. For laboratory-measured parameters, including F^−^, NH_4_^+^-N, NO_3_^−^-N, and COD_Mn_, reagent blanks, calibration standards, and duplicate samples were included where applicable. Analytical results were evaluated against the quality-control requirements specified in the corresponding national or industry standards.

### 2.4. DNA Extraction and High-Throughput Sequencing

Genomic deoxyribonucleic acid (DNA)was extracted from water-filter membranes and sediment samples using the NucleoSpin 96 Soil Kit (Macherey-Nagel, Beijing, China) according to the manufacturer’s instructions. DNA concentration and purity were assessed by 1% agarose gel electrophoresis, and the samples were diluted with sterile water to 1 ng/µL. The V3–V4 hypervariable regions of the bacterial 16S rRNA gene were amplified using primers 341F (5′-CCTACGGGAGGCAGCAG-3′) and 806R (5′-GGACTACHVGGGTWTCTAAT-3′). Each 15 µL polymerase chain reaction (PCR) mixture contained Phusion^®^ High-Fidelity PCR Master Mix (New England Biolabs, Ipswich, MA, USA), 0.2 µM of each primer, and approximately 10 ng of template DNA. Thermal cycling consisted of initial denaturation at 98 °C for 1 min; 30 cycles of 98 °C for 10 s, 50 °C for 30 s, and 72 °C for 30 s; and a final extension at 72 °C for 5 min. PCR products were pooled at equimolar concentrations and purified using the QIAquick Gel Extraction Kit (Qiagen, Beijing, China). Sequencing libraries were prepared using the NEBNext^®^ Ultra™ II DNA Library Prep Kit (New England Biolabs, Suzhou, China) and quantified using Qubit (Thermo Fisher Scientific, Waltham, MA, USA) and quantitative polymerase chain reaction (qPCR). The libraries were sequenced on an Illumina NovaSeq 6000 platform (Illumina, San Diego, CA, USA) to generate 250 bp paired-end reads (PE250).

Raw sequencing data were processed using QIIME 2 version 2022.2 [19]. Sequence quality control, error correction, denoising, paired-end read merging, and chimera removal were performed using the DADA2 (202202) plugin to generate amplicon sequence variants (ASVs) [20]. All DADA2 parameters were kept at their default settings, including the maximum expected error thresholds, except that the truncation lengths for the forward and reverse reads were truncated at 260 bp and 240 bp, respectively. Chimeric sequences were identified and removed using the default chimera-handling procedure implemented in DADA2. ASVs with fewer than five total reads across the entire dataset were subsequently removed from downstream analyses to reduce the influence of extremely low-abundance features that may represent sequencing noise, PCR artefacts, or unstable signals, particularly in diversity, βNTI-based assembly, and co-occurrence network analyses. Taxonomic classification of ASVs was performed against the SILVA 138.1 database [21]. Alpha- and β-diversity analyses were conducted using a rarefied ASV table, whereas taxonomic composition was calculated based on relative abundance. The βNTI-based null-model analysis was performed using the filtered and normalised ASV table. All sequencing and bioinformatics analyses were performed by Novogene Bioinformatics Technology Co., Ltd. (Beijing, China).

### 2.5. Data Analysis and Graphing

Alpha-diversity indices were calculated for each season and habitat using the vegan package in R (4.4.3). Differences in α-diversity and environmental variables among groups were evaluated using the Kruskal–Wallis test followed by Dunn’s post hoc test; the results were displayed as bar plots with significance annotations using the ggpubr package. Non-metric multidimensional scaling (NMDS), based on Bray–Curtis dissimilarities, was used to visualise patterns in bacterial community composition among seasons and habitats. Permutational multivariate analysis of variance (PERMANOVA) was then performed using the adonis2 function in vegan with 999 permutations to test for differences in community composition. Analysis of similarities (ANOSIM) was used as a complementary rank-based test of group separation. Differentially abundant taxa between habitats were identified using the Kruskal–Wallis test followed by Dunn’s post hoc test, whereas taxa associated with season and habitat were identified using linear discriminant analysis effect size (LEfSe). LEfSe was applied to taxonomic abundance tables collapsed at multiple annotated levels, including phylum, class, order, family, and genus. High-dimensional biomarkers indicative of seasonal or habitat groups were identified using the microeco package with an linear discriminant analysis (LDA) threshold > 4.0 [22]. Before conducting ordination analysis, variance inflation factors (VIFs) were computed to eliminate strongly correlated environmental variables (VIF > 10). Spearman correlation analysis was performed to evaluate associations between dominant or differentially abundant taxa and physicochemical parameters, with findings visualised as heatmaps using the pheatmap package. It should be noted that these correlations were interpreted as statistical associations rather than direct evidence of causal relationships.

Co-occurrence networks were constructed to explore potential associations among bacterial taxa. Only significant Spearman correlations with |r| > 0.6 and *p* < 0.05 were included. Network topological properties, including the numbers of nodes and edges, modularity, average path length, connectedness, and network density, were calculated using the igraph package, and the networks were visualised in Gephi (0.10.1, http://gephi.github.io/, accessed on 25 November 2025). Community assembly processes were evaluated using a βNTI-based null-model framework. First, the β-mean nearest taxon distance (βMNTD) was calculated to quantify the phylogenetic turnover between pairs of bacterial communities. βNTI was then calculated by comparing the observed βMNTD values with a null distribution generated by randomising taxa across the phylogenetic tree 999 times. βNTI values greater than +2 were interpreted as heterogeneous selection, whereas βNTI values lower than −2 were interpreted as homogeneous selection. Pairwise comparisons with |βNTI| ≤ 2 were interpreted as stochastic processes sensu lato [23]. Because RCBray was not applied in this study, different stochastic processes, such as dispersal limitation, homogenizing dispersal, ecological drift, or undominated processes, were not further separated.

## 3. Results

### 3.1. Temporal Variation in Physicochemical Characteristics of Water and Sediments

Sediment physicochemical parameters were generally more stable across seasons than those in the water column (Table S2; Figure S1). Sediment pH remained within a relatively narrow alkaline range of 8.02–8.74. TP and TN also showed only slight seasonal variation, ranging from 0.032% to 0.080% and from 0.029% to 0.235%, respectively. Although NH_4_^+^-N, EC, SAL, F^−^, and NO_3_^−^-N differed among seasons, the overall seasonal variation in sediments was weaker than that in the water column.

By contrast, reservoir water exhibited more pronounced seasonal variation (Table S3; Figure S2). Water temperature differed significantly among seasons. The pH increased from 7.84 ± 0.21 in April to 9.09 ± 0.29 in July and 9.11 ± 0.26 in October (*p* < 0.001). NH_3_-N followed a similar increasing trend and reached its highest concentration in October. COD_Mn_ increased from 3.08 mg/L in April to 6.78 mg/L in July, an increase of approximately 120%, indicating a marked summer rise in organic matter-related oxygen demand. TN concentrations ranged from 0.15 to 3.40 mg/L, and 37.5% of the October water samples exceeded 2.0 mg/L, although the overall seasonal difference in TN was not statistically significant. These results indicate that physicochemical conditions in the water column were more seasonally variable than those in sediments, particularly with respect to WT, pH, NH_3_-N, COD_Mn_, and TN.

### 3.2. Habitat and Season-Specific Patterns of Bacterial Community Composition

**Figure 2:**
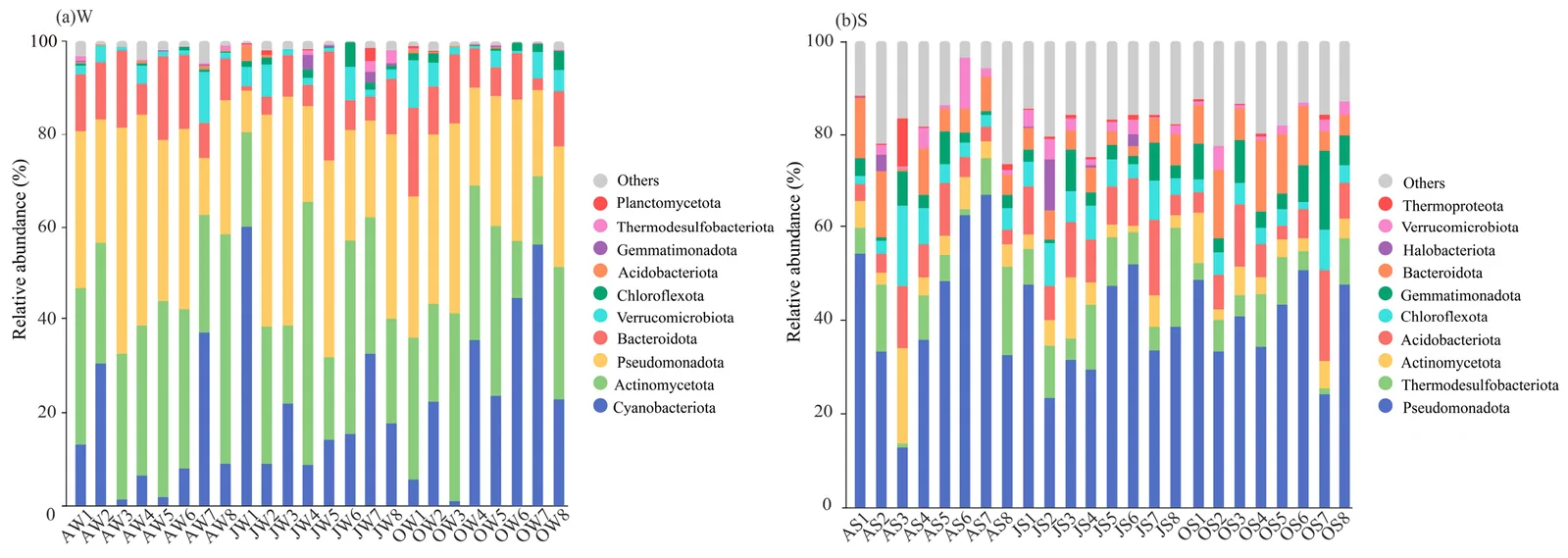
Phylum-level composition of bacterial communities in water (**a**) and sediment (**b**) samples across seasons. Numbers 1–8 represent different sampling reservoirs. A, J, and O represent April, July, and October, respectively; W and S represent water and sediment samples, respectively.

A total of 44,712 ASVs were identified in the water and sediment samples; shared and habitat-specific ASVs are shown in Figure S3. At the phylum level, the bacterial communities displayed both shared dominance and habitat-specific enrichment (Figure 2). Pseudomonadota and Acidobacteriota were dominant in both habitats, whereas Cyanobacteriota was enriched primarily in water and Thermodesulfobacteriota and Chloroflexota were more prominent in sediments. Seasonal variation was more apparent in water communities, particularly for Cyanobacteriota and other plankton-associated phyla, whereas sediment communities exhibited a more stable phylum-level composition across seasons.

To provide greater taxonomic resolution, community composition was also summarised at the genus level (Figure S4). Compared with the phylum-level pattern, genus-level composition showed greater sample-specific heterogeneity. A large proportion of sequences were assigned to low-abundance or unclassified genera, indicating substantial fine-scale taxonomic diversity in both water and sediment communities. Dominant or frequently detected genera included *Thiobacillus*, *Hydrogenophaga*, *Luteolibacter*, and *Methanothrix*, together with several unclassified groups affiliated with Bacteroidetes vadinHA17, Gemmatimonadaceae, Steroidobacteraceae, Nitrososphaeraceae, SC-I-84, and S085.

### 3.3. The Spatiotemporal Dynamics of Bacterial Diversity and Composition

**Figure 3:**
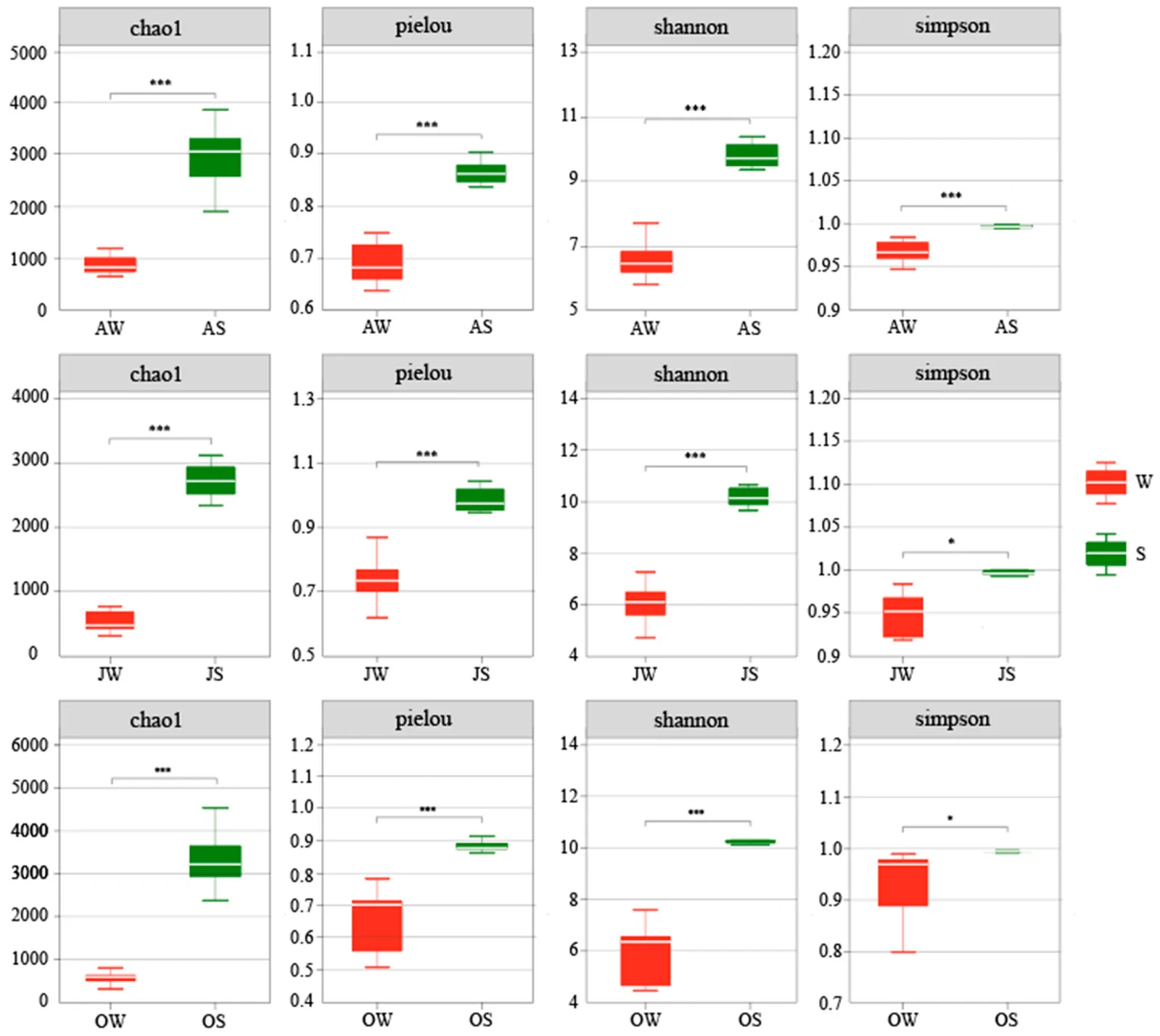
Alpha-diversity indices of bacterial communities in water and sediment samples across seasons. * *p* < 0.05, and *** *p* < 0.001.

After quality filtering and normalisation, 3,818,880 high-quality reads with an average length of 420 bp were obtained from 24 water and 24 sediment samples collected from eight reservoirs over three seasons. The mean sequencing depth was 81,082 reads per water sample and 78,038 reads per sediment sample. Bacterial α-diversity differed significantly between water and sediment across seasons (Figure 3; *p* < 0.05). Sediment communities were markedly more diverse than water communities, with mean Chao1, Pielou’s evenness, Shannon, and Simpson indices of 3063.12, 0.869, 9.98, and 0.996, respectively, compared with 748.38, 0.668, 6.25, and 0.942 in water.

Statistical Analysis of Metagenomic Profiles (STAMP) analysis (Figure S5) revealed substantial differences in bacterial composition between water and sediment. Pseudomonadota was the predominant phylum in both habitats and was significantly enriched in sediments (*p* < 0.05). Water communities had higher relative abundances of Actinomycetota and Cyanobacteriota, with the latter being nearly absent from sediments (*p* < 0.001). Conversely, sediments contained significantly higher relative abundances of benthic-associated phyla, including Thermodesulfobacteriota, Acidobacteriota, and Chloroflexota (*p* < 0.001).

**Figure 4:**
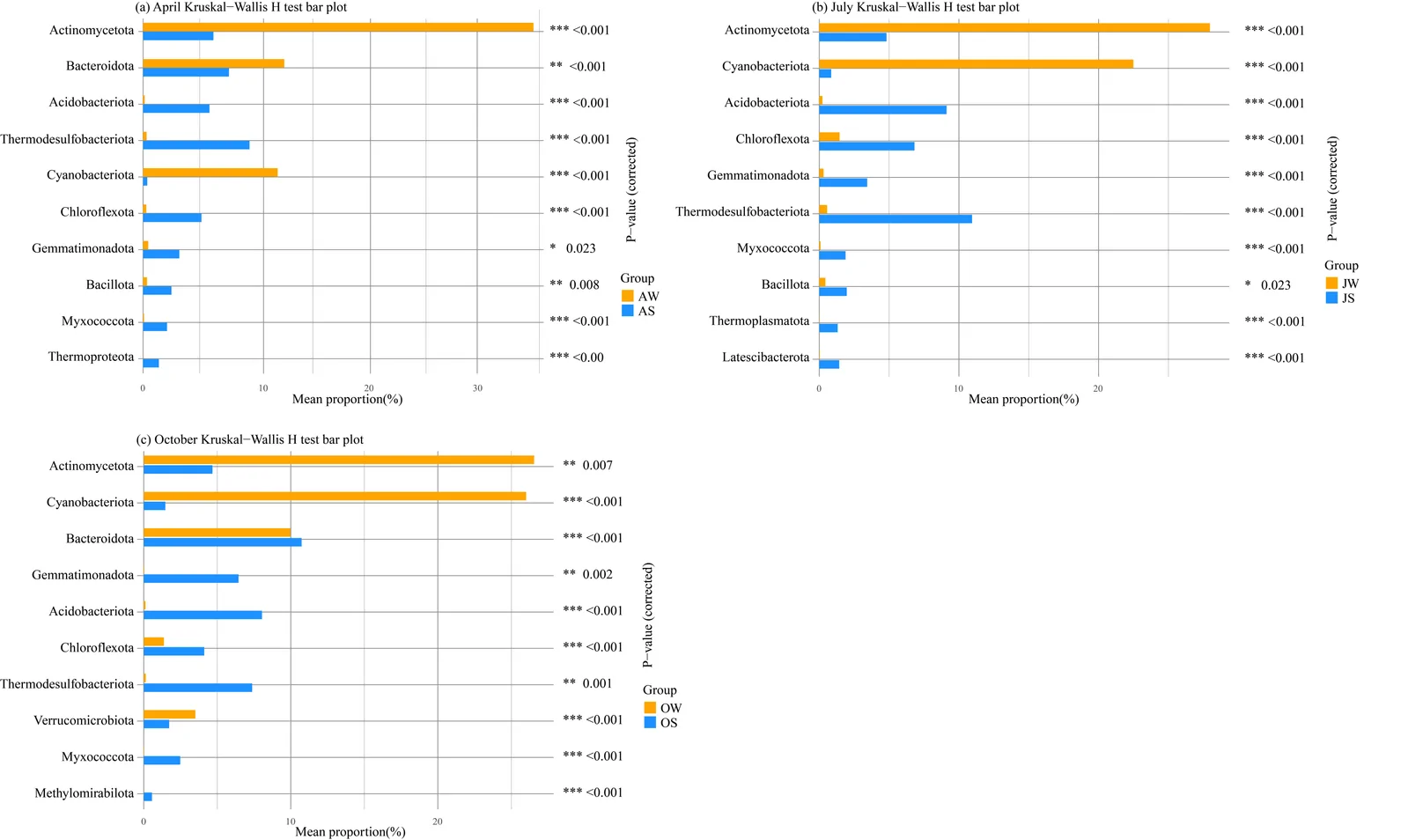
Results of Kruskal–Wallis tests comparing dominant bacterial phyla between water and sediment samples. Significance levels are indicated as follows: * *p* < 0.05, ** *p* < 0.01, and *** *p* < 0.001.

STAMP and Kruskal–Wallis analyses further confirmed significant differences in the relative abundance of dominant phyla between water and sediment samples (Figure 4; Figure S5). Cyanobacteriota and Actinomycetota were significantly enriched in water, whereas Thermodesulfobacteriota, Acidobacteriota, Chloroflexota, and Gemmatimonadota were more abundant in sediments.

**Figure 5:**
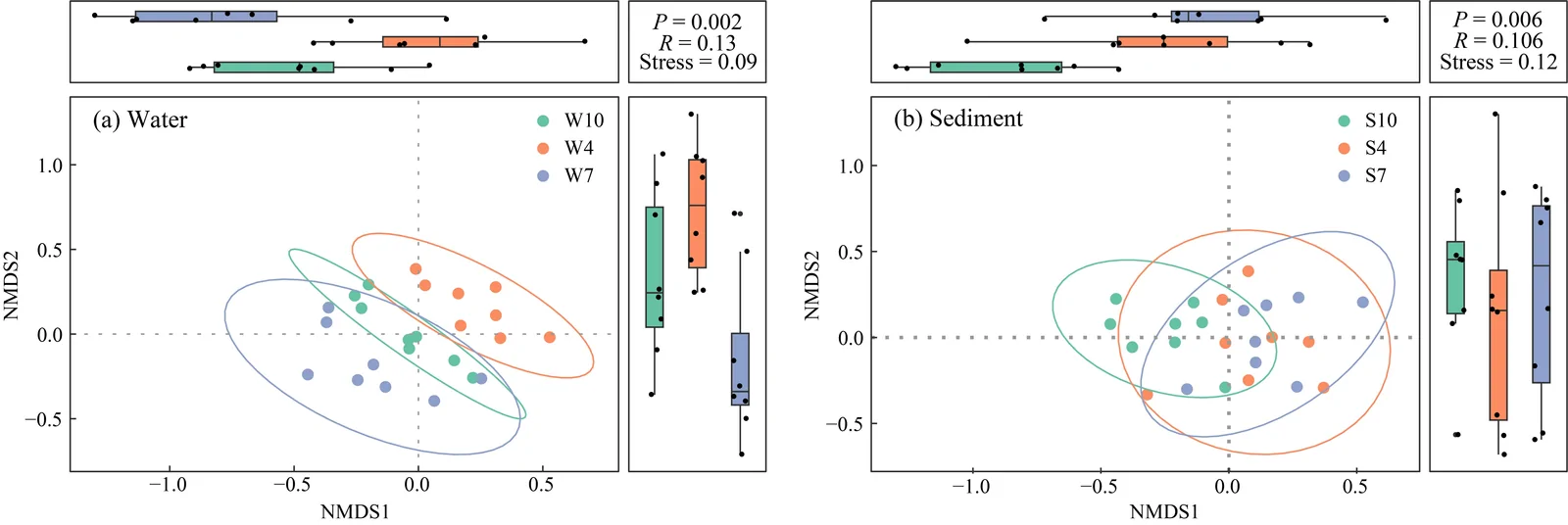
Non-metric multidimensional scaling (NMDS) ordinations of bacterial community composition.

β-Diversity was evaluated by NMDS based on Bray–Curtis dissimilarities, and seasonal differences in bacterial community composition were further assessed using ANOSIM and pairwise PERMANOVA (Table S4). In water, the NMDS ordination showed clear seasonal separation in Figure 5, particularly between April and July along the NMDS1 axis, and ANOSIM indicated significant overall seasonal differentiation (*p* = 0.002, R = 0.13, stress = 0.09). Pairwise PERMANOVA further showed that bacterial communities in April differed significantly from those in July and October (AW vs. JW: R^2^ = 0.1139, *p* = 0.001; AW vs. OW: R^2^ = 0.0968, *p* = 0.013), whereas the difference between July and October was weaker and not statistically significant (JW vs. OW: R^2^ = 0.0897, *p* = 0.055). In sediments, although ANOSIM also indicated significant overall seasonal differentiation (*p* = 0.006, R = 0.106, stress = 0.12), the NMDS ordination showed greater overlap among seasons. Pairwise PERMANOVA indicated no significant difference between April and July sediments (AS vs. JS: R^2^ = 0.0581, *p* = 0.877), whereas October sediments differed significantly from both April and July sediments (AS vs. OS: R^2^ = 0.0948, *p* = 0.003; JS vs. OS: R^2^ = 0.0927, *p* = 0.003). Overall, planktonic bacterial communities exhibited stronger seasonal turnover than sediment communities. Sediment communities remained relatively stable from April to July but shifted distinctly in October. All NMDS stress values were below 0.15, indicating good ordination fits for ecological community data. On the basis of these habitat- and season-related patterns, LEfSe analysis was subsequently conducted to identify differentially abundant taxa contributing to community differentiation.

### 3.4. Differentially Abundant Bacterial Taxa Across Habitats and Seasons

**Figure 6:**
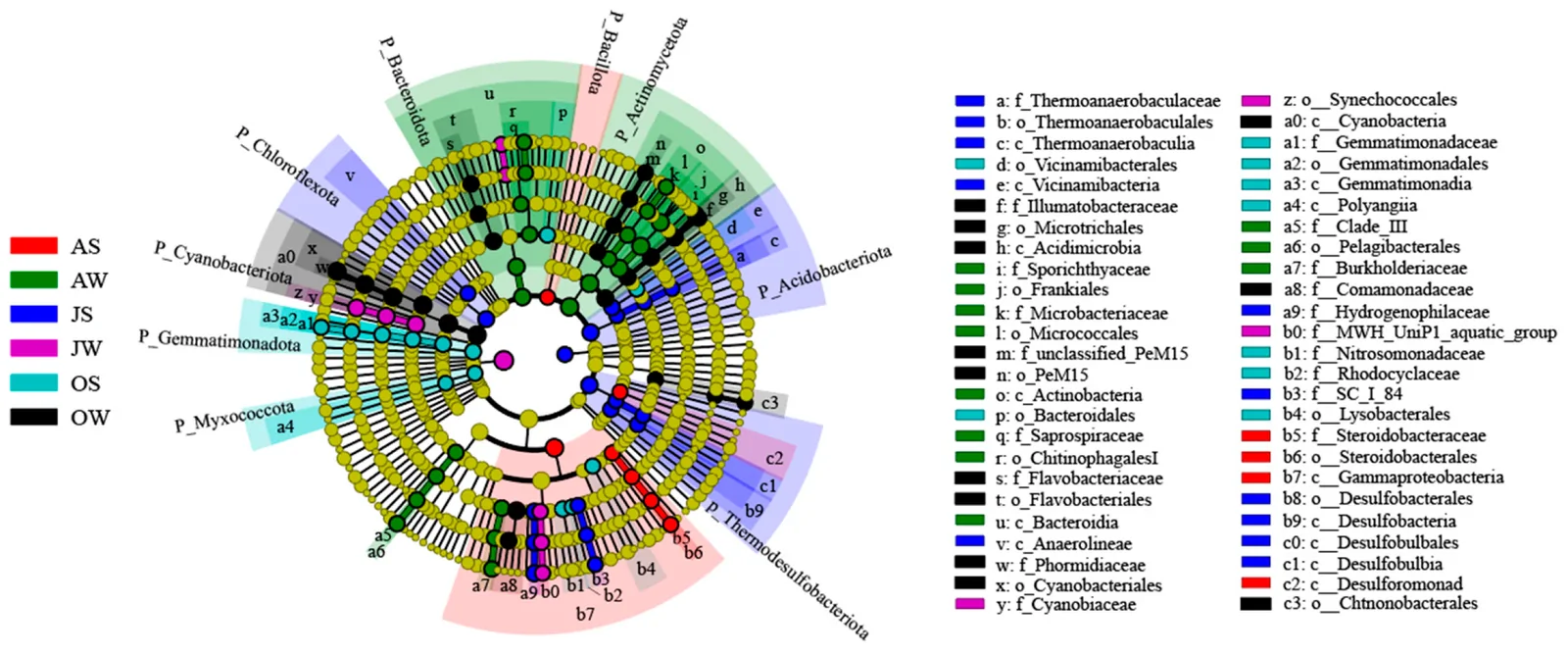
Differentially abundant bacterial taxa identified by LEfSe analysis across habitats and seasons.

Linear discriminant analysis effect size (LEfSe), with an LDA threshold of 4, was used to identify bacterial taxa associated with habitat and season (Figure 6). In total, 50 taxa differed significantly among seasons or habitats in water and sediment. Seasonal succession was evident in water, with distinct indicator taxa enriched in different seasons. In April, *Actinomycetota* and its subordinate orders, including Micrococcales and Frankiales, were significantly enriched. In July, elevated temperatures and high light availability were associated with enrichment of the unicellular cyanobacterial order Synechococcales. By October, enrichment had shifted towards larger filamentous Cyanobacteriota, represented mainly by Phormidiaceae and Cyanobacteriotales.

Differentially abundant taxa in sediments were primarily associated with anaerobic and benthic bacterial groups. Desulfobacterales (Thermodesulfobacteriota) were enriched in April, whereas Desulfobulbales (Thermodesulfobacteriota) were enriched in July. In October, Gemmatimonadota (Gemmatimonadales) and Myxococcota were the predominant differentially abundant taxa.

### 3.5. Environmental Associations and Microbial Community Assembly Processes

**Figure 7:**
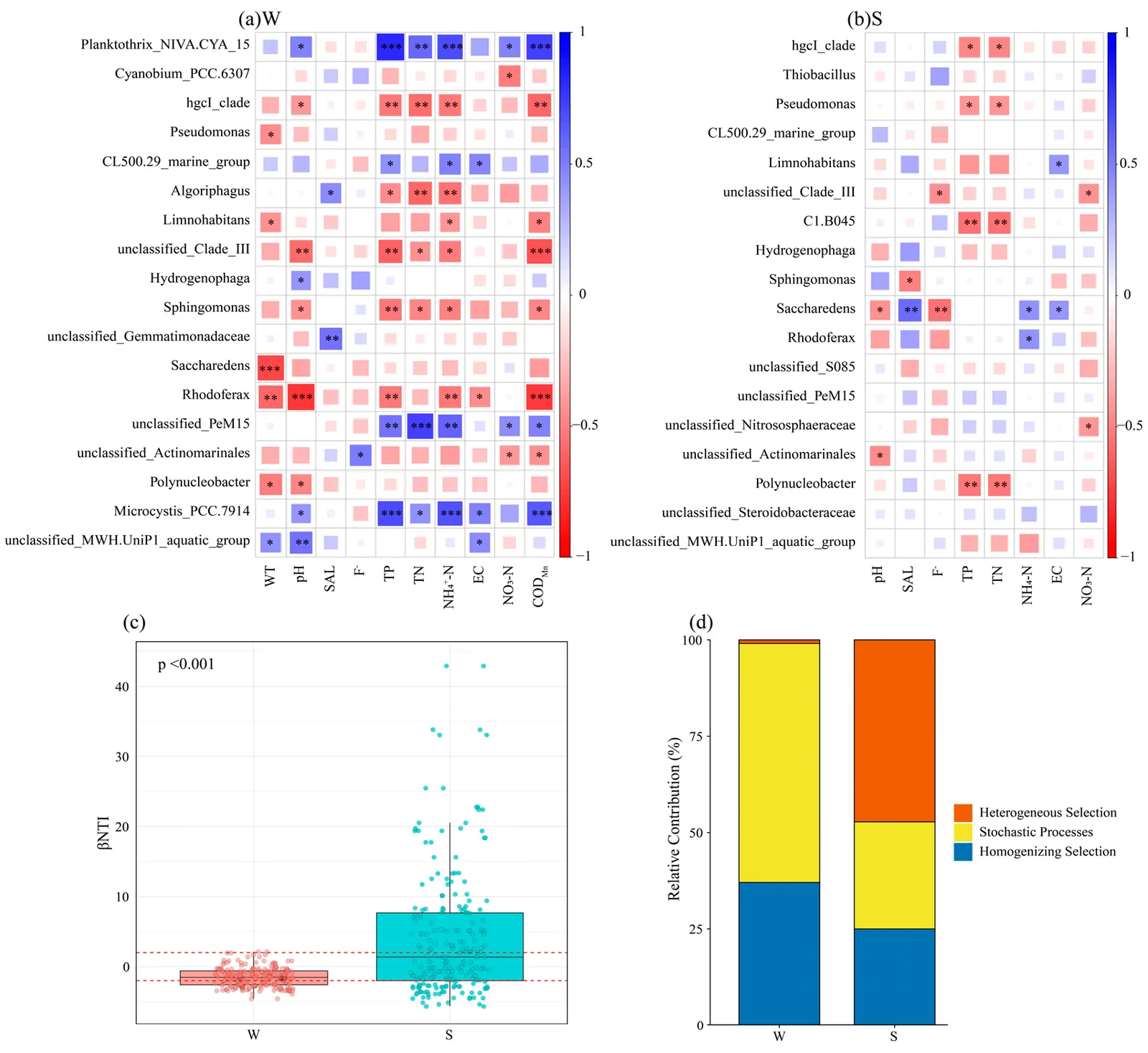
Environmental associations and community assembly processes in water and sediment bacterial communities. Heatmaps show Spearman correlations between environmental variables and dominant bacterial taxa in water (**a**) and sediments (**b**). βNTI distributions are shown in different habitats (**c**), and the relative contributions of homogeneous selection, heterogeneous selection, and stochastic processes sensu lato are shown in (**d**). *: *p* < 0.05, **: *p* < 0.01, ***: *p* < 0.001.

Spearman correlation analysis revealed distinct taxon–environment associations in water and sediment samples (Figure 7a,b). Significant correlations were more frequent in water and mainly involved WT, pH, TP, TN, NH_3_-N, NO_3_^−^-N, COD_Mn_, EC, and SAL. Planktothrix_NIVA-CYA_15, Microcystis_PCC-7914, and unclassified_PeM15 were positively correlated with nutrient- and organic matter-related variables, including TP, TN, NH_3_-N, NO_3_^−^-N, and COD_Mn_ (ρ = 0.407–0.818, *p* < 0.05). By contrast, *Rhodoferax*, *Algoriphagus*, hgcI_clade, and *Sphingomonas* were negatively correlated with several nitrogen- or organic matter-related variables and, in some cases, with pH or temperature (ρ = −0.777 to −0.457, *p* < 0.05).

Sediment samples showed fewer significant correlations, which mainly involved NO_3_^−^-N, NH_3_-N, pH, salinity, TP, and TN. NO_3_^−^-N was negatively correlated with *Algoriphagus* and hgcI_clade but positively correlated with unclassified_PeM15, whereas NH_3_-N showed contrasting associations with *Microcystis_PCC-7914* and *Rhodoferax*. Overall, water bacterial communities were more closely associated with seasonal physicochemical variation, whereas sediment communities exhibited fewer and more taxon-specific environmental associations.

A βNTI-based null-model analysis was used to evaluate the relative contributions of deterministic selection and stochastic processes sensu lato to bacterial community assembly. Most βNTI values, particularly those for water samples, fell between −2 and +2 (Figure 7c), indicating generally weak deterministic selection and an important role for stochastic processes in the broad sense. Water bacterial communities were predominantly associated with stochastic processes, whereas sediment communities showed a greater contribution from deterministic processes, particularly heterogeneous selection (Figure 7d). These findings indicate that planktonic bacterial communities were more strongly influenced by stochastic turnover, whereas sediment bacterial communities were more strongly structured by habitat heterogeneity and environmental selection.

### 3.6. Co-Occurrence Network Patterns and Topologically Important Taxa

**Figure 8:**
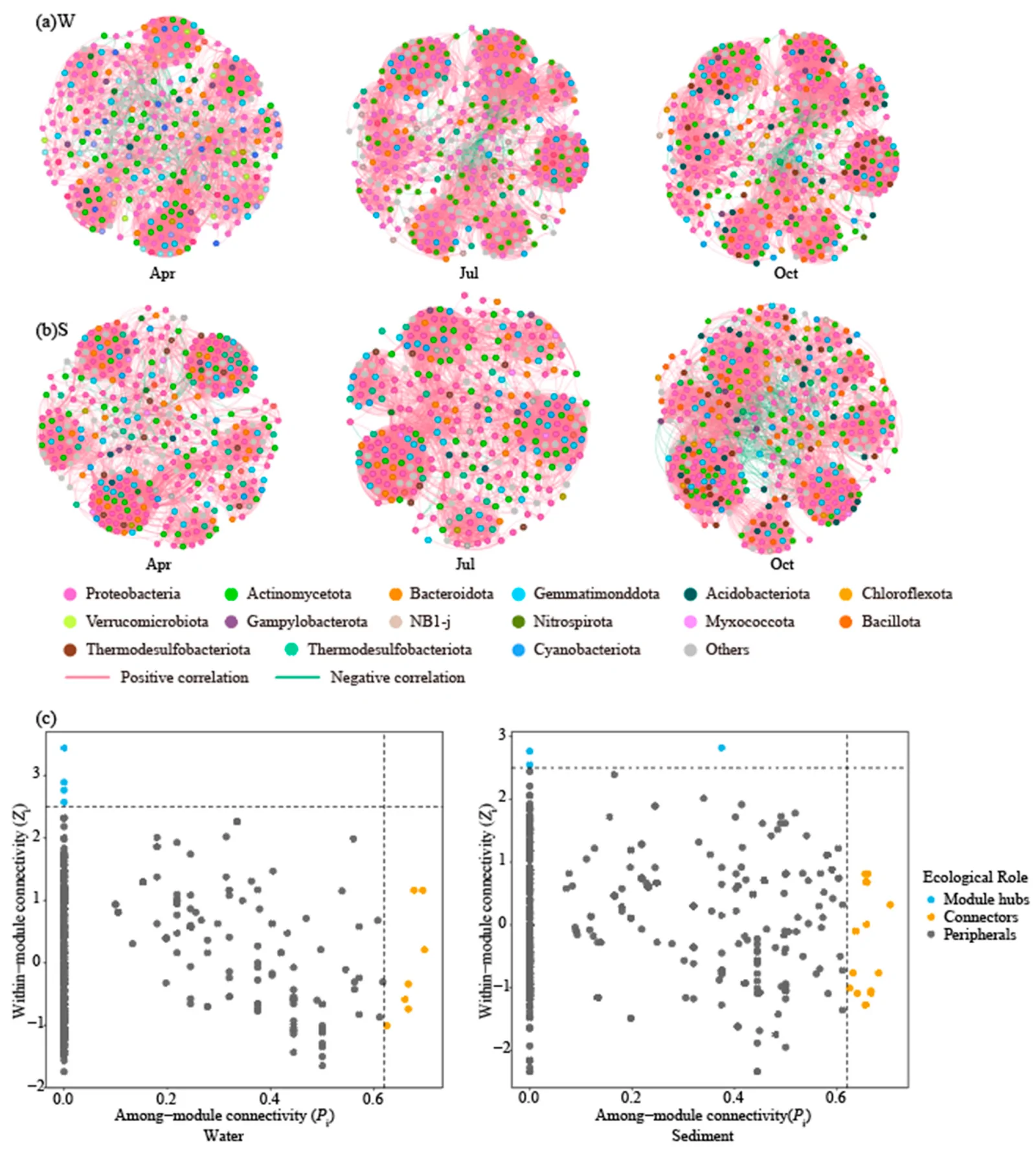
Co-occurrence network patterns and Zi-Pi analysis of bacterial communities in water and sediments. Networks show potential bacterial co-occurrence associations in water (**a**) and sediment (**b**) samples across seasons. Zi–Pi plots show the topological roles of taxa in water and sediment networks (**c**). Nodes represent bacterial taxa, and edges represent significant Spearman correlations.

Co-occurrence network and Zi-Pi analyses were performed to explore potential associations among taxa and to identify topologically important taxa (Figure 8). The network analysis revealed clear habitat- and season-related differences in bacterial association patterns (Table 1). Overall, sediment networks were more complex than water networks, with higher connectivity and more associations among taxa. This pattern suggests that sediments may provide more heterogeneous and stable microhabitats, thereby supporting more highly connected bacterial assemblages and potentially greater functional redundancy.

Seasonal differences were particularly evident in July. Both water and sediment networks showed increased complexity in July, and this pattern was especially pronounced in sediments. The July sediment network exhibited higher connectivity and a greater proportion of positive correlations, indicating stronger potential co-occurrence associations among bacterial taxa under summer conditions. This may be related to increased nutrient availability and organic matter inputs during the summer period.

These network patterns suggest that bacterial communities, especially in sediments, may become more connected and structurally complex under nutrient-enriched summer conditions. Higher connectivity and modular organisation may contribute to community stability and robustness by distributing associations across multiple taxa and modules. However, because the networks were based on Spearman correlations, positive edges should be interpreted as potential co-occurrence associations rather than direct cooperative interactions.

### 3.7. The Relationship Between Environmental Factors and the Pattern of Bacterial Diversity

**Figure 9:**
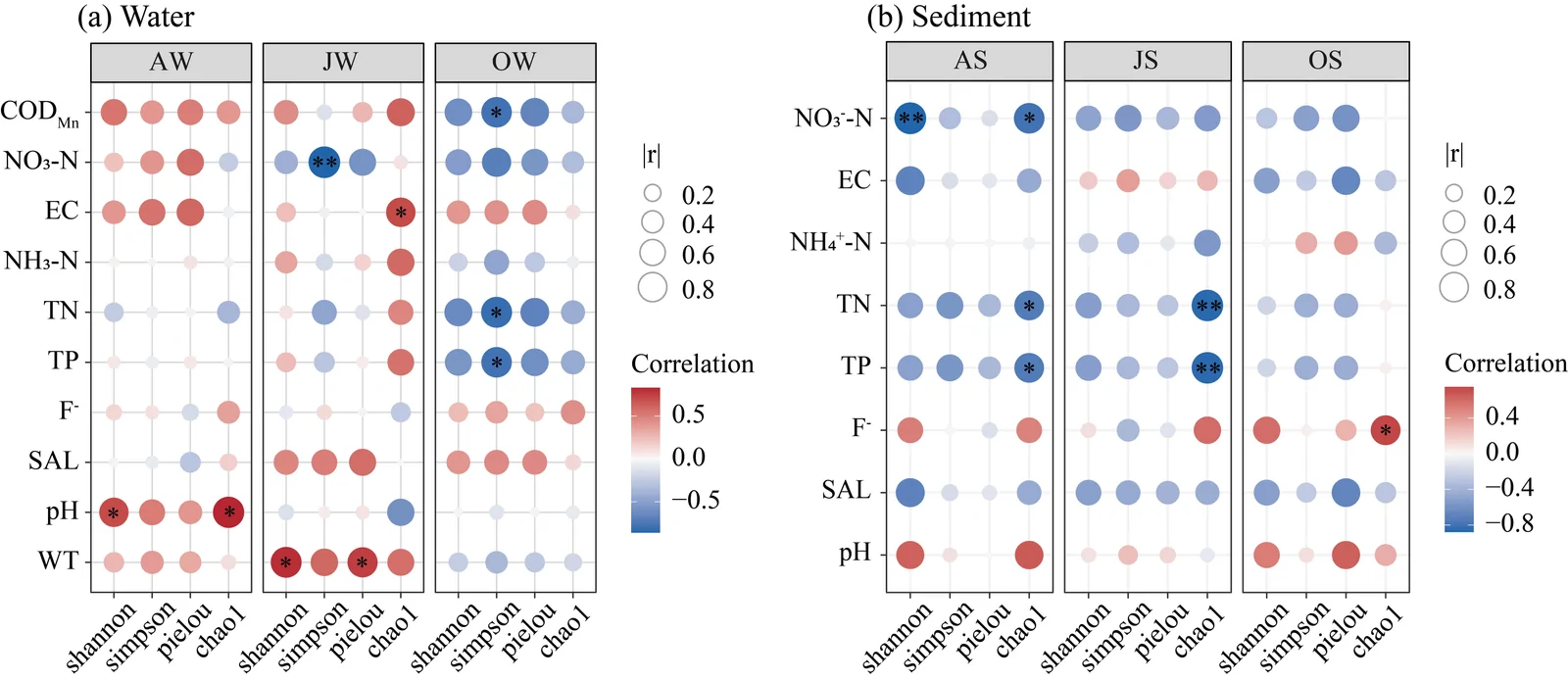
Analysis of α-diversity of bacterial communities in water samples and sediments and environmental factors. *: *p* < 0.05, **: *p* < 0.01.

Correlation analyses were conducted to examine associations between environmental variables and bacterial α-diversity indices (Figure 9). In water (Figure 9a), April pH was significantly and positively correlated with the Shannon and Chao1 indices (*p* < 0.05). In July, water temperature was significantly and positively correlated with the Shannon and Pielou’s indices, whereas NO_3_^−^-N was strongly and negatively correlated with the Simpson index (*p* < 0.01). In October, COD_Mn_, TN, and TP were significantly and positively correlated with the Simpson index.

Sediments exhibited distinct correlation patterns (Figure 9b). In April, NO_3_^−^-N was strongly and negatively correlated with the Shannon index, whereas TN and TP were negatively correlated with Chao1. In July, the negative associations of TN and TP with Chao1 became stronger and highly significant. In October, only F^−^ was significantly and positively correlated with Chao1.

## 4. Discussion

### 4.1. Habitat Differentiation of Bacterial Communities and Seasonal Heterogeneity

The contrasting bacterial diversity and composition between of water and sediments reflect the distinct ecological roles of these two reservoir habitats. Compared with the water column, sediments provide more stable attachment surfaces, accumulate more organic matter, and contain stronger physicochemical gradients, thereby creating diverse ecological niches for bacterial colonisation [24]. These characteristics may explain the greater bacterial diversity of sediments and the enrichment of sediment-associated taxa, including Thermodesulfobacteriota, Acidobacteriota, Chloroflexota, and Gemmatimonadota, in sediment communities. In contrast, the water column is more directly exposed to seasonal changes in temperature, light availability, precipitation, evaporation, and nutrient inputs, making planktonic bacterial communities more responsive to short-term environmental variation [25]. This interpretation is consistent with the α- and β-diversity results, which showed higher diversity and weaker seasonal turnover in sediments but stronger seasonal restructuring in water communities.

The habitat-specific patterns observed in this study are broadly consistent with previous findings from freshwater reservoirs and lakes, where sediments often act as microbial reservoirs and water communities show stronger temporal variability [26]. However, the relatively weak seasonal turnover of sediment bacterial communities may be particularly relevant to reservoirs in arid and semi-arid regions. In the study area, limited annual precipitation, strong evaporation, weak inflow, and reduced hydrodynamic exchange may help maintain stable sediment microhabitats and reduce frequent sediment resuspension. These conditions may buffer sediment bacterial communities against short-term seasonal disturbances [27]. By contrast, concentrated summer rainfall may introduce external nutrients, particulate matter, and microorganisms into reservoir water, thereby being associated with the seasonal reorganisation of planktonic bacterial communities.

### 4.2. The Assembly Mechanism of Bacterial Communities in Different Habitats

In recent years, clarifying the fundamental mechanisms of community assembly has been a central topic in microbial ecology. Deterministic and stochastic mechanisms are believed to jointly influence microbial community aggregation, while their respective contributions are still contested [23,28]. In the reservoirs of arid and semi-arid regions, water-column variables in water, including pH, WT, COD_Mn_, TP, and NH_3_-N, exhibited pronounced seasonal fluctuations and may be associated with changes in community composition and assembly patterns [29]. Null model analyses were utilised to assess the relative significance of deterministic and stochastic processes in bacterial communities across various habitats. We discovered that communities in both water and sediments were influenced by a blend of deterministic and stochastic processes; however, their relative importance differed markedly between habitats (Figure 7). The βNTI values of water bacterial communities were mainly distributed between −2 and +2, suggesting that deterministic selection was relatively weak and that stochastic processes sensu lato may have played an important role. However, because βNTI alone cannot distinguish among different stochastic processes, such as dispersal limitation, homogenizing dispersal, and ecological drift, these specific processes were not separately inferred [30].

Compared with water communities, sediment bacterial communities exhibited a stronger deterministic assembly signal, with heterogeneous selection making a greater contributing more prominently to community turnover. This pattern is ecologically plausible because sediments contain pronounced microscale gradients in redox conditions, organic matter, nutrient availability, and pH [31], thereby creating spatially distinct ecological niches and favouring different bacterial taxa across reservoirs and seasons [32]. Homogeneous selection may also occur where sediment physicochemical conditions converge, but it appeared to play a secondary role in the present study. Previous studies have similarly shown that long-term environmental filtering and hydrological regulation can strengthen deterministic assembly in anthropogenically modified aquatic ecosystems [33].

The relatively limited restricted exchange among sediment microhabitats may further enhance the influence of local environmental filtering, because lower physical connectivity can reduce community homogenisation relative to the water column [34]. Consistent with this interpretation, several sediment-associated taxa were significantly correlated with TN, TP, pH, and salinity, all of which can influence resource availability, metabolic activity, and taxon-specific niche suitability [35]. Nitrogen and phosphorus availability may regulate microbial growth and competitive relationships [36], whereas pH can affect nutrient solubility, membrane energetics, and enzyme activity, thereby selecting for taxa with different physiological tolerances [37]. These environmental gradients may therefore contribute to the stronger heterogeneous-selection signal observed in sediments.

### 4.3. Ecological Implications and Limitations of Bacterial Co-Occurrence Networks

Co-occurrence network analysis provided an exploratory view of potential bacterial association patterns across habitats and seasons [38,39]. Network complexity peaked in July in both habitats, particularly in sediments, as indicated by higher numbers of nodes and edges, greater average degree and clustering coefficients, and a larger proportion of positive correlations. This pattern may reflect shared responses of bacterial taxa to summer environmental conditions, including warmer temperatures, increased microbial activity, nutrient enrichment, organic matter inputs, and episodic hydrological disturbance [40]. In arid and semi-arid reservoirs, concentrated summer rainfall may also introduce catchment-derived particles, nutrients, and microorganisms into reservoir systems [3]. However, because rainfall, inflow, and external nutrient loads were not measured, these processes should be regarded as plausible explanations rather than confirmed drivers.

Sediment networks were generally more densely connected than water networks, which may be related to the relatively persistent and heterogeneous nature of sediment habitats [41]. Sediments can accumulate organic matter, nutrients, particles, and microorganisms, while maintaining microscale gradients in oxygen availability, redox conditions, and substrate quality. These characteristics may create diverse ecological niches and support more complex potential associations among bacterial taxa. In contrast, water communities are more directly exposed to short-term variation in temperature, mixing, hydrological connectivity, and nutrient inputs, which may contribute to greater seasonal instability in network structure. Previous studies have also shown that seasonal succession [42], spatial heterogeneity [43], and disturbance can alter modify microbial network topology in aquatic ecosystems [44].

Temperature and nutrient availability may partly explain the greater network complexity observed in July. Nitrogen and phosphorus are important resources regulating microbial growth and community composition in lakes and reservoirs [45], and prolonged water residence may enhance particle retention and nutrient accumulation, particularly at the sediment–water interface [46]. Higher temperature can further stimulate microbial metabolism and resource turnover [47]. In this study, TN and TP were associated with Pseudomonadota, *Polynucleobacter*, and the *hgcI clade*, while relatively high NH_4_^+^-N concentrations in July sediments may have been associated with nutrient-responsive taxa [48]. In addition, algal detritus, catchment-derived particles, and plant residues may accumulate on sediment surfaces [49] and increase substrate heterogeneity [50]. Nevertheless, the high proportion of positive correlations in July should not be interpreted as evidence of direct cooperation among taxa. Positive edges may also arise from shared environmental preferences, indirect associations, or compositional effects [51]. Similarly, greater clustering and connectivity should be interpreted as indicators of network structural complexity rather than direct evidence of ecological stability, resilience, or functional redundancy.

Zi-Pi analysis identified several topologically important taxa. Bacteroidota included potential connector taxa in sediment networks, whereas Bacillota contributed to inter-module connectivity in water networks. These taxa may link distinct modules, although their ecological functions require further validation. Importantly, topological importance does not necessarily correspond to high relative abundance [52].

In October, sediment F^−^ concentrations were positively correlated with Chao1 richness. This association may reflect the geogenic fluoride background of Ningxia and its influence on sediment geochemistry [53]. Nevertheless, the observational design does not support a causal interpretation, and this relationship should be treated as a hypothesis requiring experimental validation [18]. From a management perspective, these findings suggest that water-quality monitoring in arid and semi-arid reservoirs should be intensified during summer, with particular attention to temperature, pH, nutrient concentrations, organic matter-related variables, and microbial community changes. In addition, the high diversity and relative stability of sediment bacterial communities indicate that sediment conditions and the potential consequences of sediment disturbance or resuspension should be incorporated into long-term reservoir assessment, particularly when operational activities may alter sediment–water exchange.

The networks were based on only eight composite samples per season and may therefore have been influenced by sample size, compositionality, sequencing depth, threshold selection, and indirect correlations [54]. Moreover, 16S rRNA gene sequencing cannot directly resolve microbial functions or activity [43]. Future studies using larger sample sizes, higher-frequency sampling, composition-aware network methods, and multi-omics approaches are needed to validate these inferred associations.

## 5. Conclusions

This study revealed clear habitat-specific and seasonal differences in bacterial communities between reservoir water and sediments in arid and semi-arid regions of northwest China. Sediments supported higher bacterial diversity, more complex co-occurrence networks, and a stronger deterministic assembly signal, whereas water communities showed stronger seasonal variation and were more closely associated with stochastic processes sensu lato and changes in temperature, pH, nutrients, and organic matter-related conditions.

Network complexity peaked in July, especially in sediments, suggesting stronger potential co-occurrence associations under summer nutrient-enriched conditions. Nutrient availability, organic matter-related variables, and geogenic ions, particularly fluoride, may jointly contribute to microbial community structure and network complexity, although the relationship between sediment fluoride and bacterial diversity remains hypothetical. These findings suggest that reservoir management should strengthen monitoring of summer nutrient pulses and organic matter inputs, while also incorporating sediment bacterial communities into long-term ecological assessment because of their potential role in sediment–water nutrient exchange and reservoir ecological stability.

## Figures and Tables

**Table 1:** Topological characteristic parameters of the co-occurrence network of bacteria in water and sediments in different seasons.

Groups	Nodes	Edges	Average Degree	Average Path Length	Positive Correlation Ratio	Modularity	Average Clustering Coefficient
W-Apr	347	4068	23.447	3.160	93.66	0.744	0.679
W-Jul	405	7005	34.593	3.21	97.40	0.759	0.756
W-Oct	331	4504	27.215	2.858	87.79	0.651	0.653
S-Apr	386	6444	33.389	3.243	98.48	0.742	0.762
S-Jul	440	10,496	47.709	3.059	99.74	0.703	0.766
S-Oct	421	7897	37.515	2.863	95.26	0.718	0.718

## Data Availability

The original contributions presented in the study are included in the article/Supplementary Material, further inquiries can be directed to the corresponding author.

## Supplementary Materials

The following supporting information can be downloaded at: https://www.mdpi.com/article/10.3390/microorganisms14081657/s1, Table S1: Information of Dam Locations. Table S2: Physical and Chemical Indicators of Sediment in Each Reservoir. Figure S1: Changes in sediment physical and chemical indicators in different seasons. Table S3: Physical and Chemical Indicators of Water in Each Reservoir. Figure S2: Changes in water physical and chemical indicators in different seasons. Figure S3: Number of bacterial ASVs in sediment (S) and overlying water (W) samples. The overlapping area represents shared ASVs between water and sediment samples. Figure S4: Genus-level composition of bacterial communities in water (a) and sediment (b) samples. Numbers 1–8 represent different sampling reservoirs. A, J, and O represent April, July, and October, respectively; W and S represent water and sediment samples, respectively. Table S4: The results of PERMANOVA analysis among different seasonal groups. Figure S5: Differences in the relative abundance of dominant bacterial phyla between water and sediment samples. (n = 24 for water; n = 24 for sediment; n = 8 per habitat in each season; Significance levels are indicated as follows: * *p* < 0.05, ** *p* < 0.01, and *** *p* < 0.001).
