# Supplementary material for: Seasonal Variation in the Assembly Mechanisms and Co-Occurrence Patterns of Bacterial Communities in Water and Sediments of Reservoirs in Arid and Semi-Arid Regions
Source: Microorganisms. 2026 Jul 29;14(8):1657. doi: 10.3390/microorganisms14081657 (PMC13515323; doi:10.3390/microorganisms14081657)
Supplement: Supplementary file 1 [file microorganisms-14-01657-s001.zip › microorganisms-4449988-supplementary.pdf]

Table S1. Information of Dam Locations.

Point	Reservoir	Longitude	Latitude	Altitude
S1	Xiazhai	105.7955	35.9589	1854.2
S2	Zhangjiazuitou	105.8603	35.8309	1830.6
S3	Sanlidian	106.0993	35.6214	2010.3
S4	Xihaizi	106.1446	35.9043	2199.8
S5	Shenjiahe	106.2805	36.1033	1620.5
S6	Naijiahe	106.3438	35.8411	1720.7
S7	Shitouyaoxian	106.6428	35.8702	1441.4
S8	Yashigou	106.7177	36.0857	1611.4

Table S2. Physical and Chemical Indicators of Sediment in Each Reservoir.

Sample ID	pH	EC (μS/cm)	SAL (‰)	F^- (mg/kg)	TN (%)	TP (%)	NH_4^+-N (mg/kg)	NO_3^--N (mg/kg)
AS1	8.30	1260	0.65	0.53	0.093	0.078	36.03	48.60
AS2	8.37	1021	0.52	0.99	0.037	0.062	61.11	5.15
AS3	8.35	783	0.39	1.11	0.043	0.055	68.46	8.94
AS4	8.53	553	0.27	1.23	0.044	0.062	39.22	6.28
AS5	8.49	434	0.21	1.06	0.077	0.064	32.08	9.36
AS6	8.60	370	0.22	1.70	0.136	0.077	40.05	15.18
AS7	8.67	454	0.26	2.89	0.048	0.062	39.21	5.13
AS8	8.74	530	0.26	1.63	0.032	0.060	34.40	5.96
JS1	8.27	1079	0.53	0.47	0.091	0.075	125.27	21.23
JS2	8.27	1472	0.74	0.92	0.041	0.061	74.93	2.77
JS3	8.50	1440	0.71	0.55	0.105	0.051	90.15	5.61
JS4	8.02	2443	1.25	0.87	0.188	0.032	166.78	18.84
JS5	8.48	415	0.20	0.58	0.074	0.068	49.63	2.92
JS6	8.54	330	0.16	0.96	0.075	0.080	91.93	3.32
JS7	8.31	805	0.39	1.32	0.029	0.064	100.68	5.00
JS8	8.35	871	0.42	1.33	0.036	0.059	72.59	4.71
OS1	8.38	606	0.30	0.844	0.090	0.075	46.55	5.57
OS2	8.32	1268	0.68	0.918	0.038	0.076	43.64	4.09
OS3	8.43	933	0.48	1.24	0.068	0.036	62.55	3.59
OS4	8.18	1250	0.64	1.35	0.235	0.050	13.09	12.98
OS5	8.54	383	0.19	1.01	0.065	0.055	71.27	6.08
OS6	8.44	443	0.22	1.12	0.093	0.063	62.40	5.68
OS7	8.31	1234	0.63	1.48	0.059	0.069	55.20	8.33
OS8	8.61	380	0.19	2.35	0.037	0.064	36.00	4.06

Note: AS, JS, and OS represent sediment samples collected in April, July, and October, respectively.

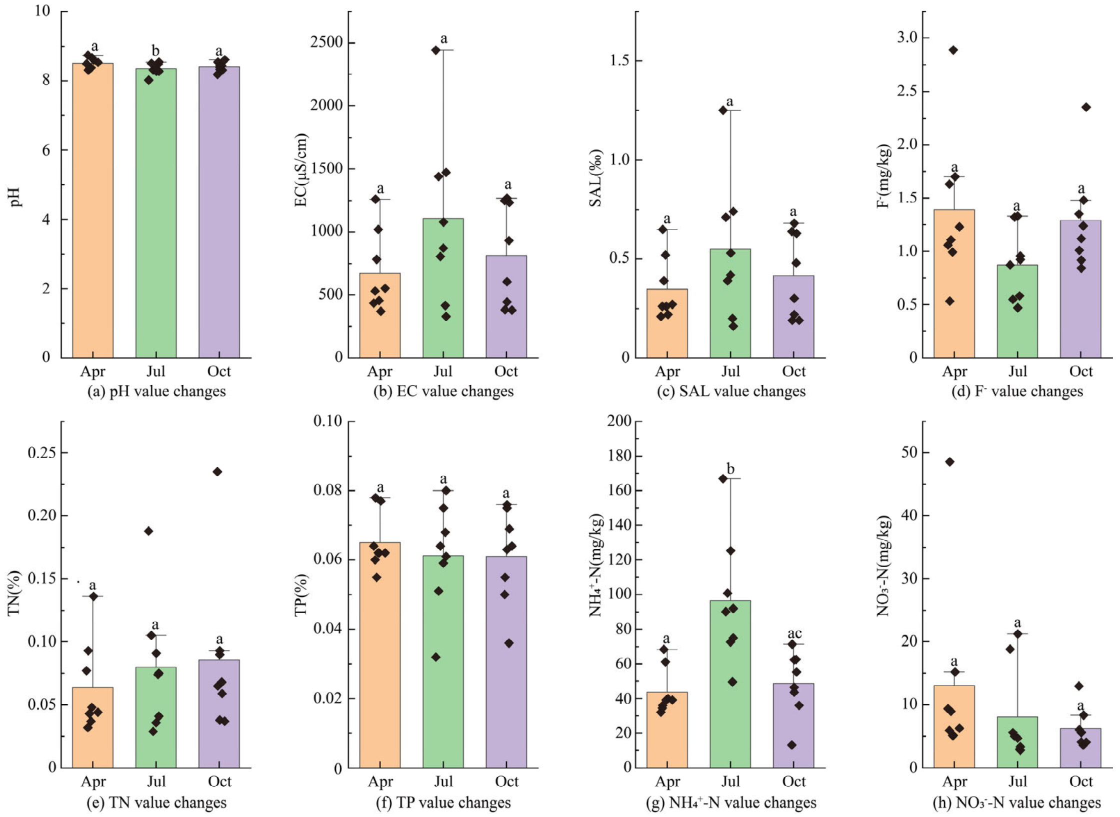

Figure S1. Changes in sediment physical and chemical indicators in different seasons.

Table S3. Physical and Chemical Indicators of Water in Each Reservoir.

Sample ID	WT (°C)	pH	EC (μS/cm)	SAL (‰)	F^- (mg/L)	TN (mg/L)	TP (mg/L)	NH_3-N (mg/L)	NO_3-N (mg/L)	COD_{Mn} (mg/L)
AW1	15.2	7.74	1641	0.97	0.831	1.475	0.12	0.52	0.22	6.22
AW2	14.1	7.76	1945	1.18	1.080	0.462	0.02	0.08	0.26	2.91
AW3	13.0	7.93	795	0.47	0.681	1.112	0.003	0.09	0.8	3.12
AW4	16.1	7.81	1232	0.79	1.280	2.984	0.02	0.34	0.24	0.76
AW5	17.5	7.67	1035	0.65	0.642	1.405	0.05	0.23	0.95	3.60
AW6	17.4	7.59	808	0.52	0.436	0.448	0.02	0.03	0.52	1.08
AW7	18.0	7.97	2436	1.47	1.412	0.153	0.02	0.03	0.33	1.83
AW8	17.2	8.26	1242	0.76	1.073	0.481	0.04	0.22	0.29	5.31
JW1	26.9	9.02	1462	0.68	0.654	2.105	0.13	0.72	0.39	11.78
JW2	30.3	9.25	2296	1.09	1.121	0.703	0.03	0.16	0.18	5.37
JW3	24.8	9.33	857	0.39	0.654	0.898	0.06	0.43	0.19	5.02
JW4	30.7	8.69	1778	0.81	1.443	0.579	0.03	0.33	0.03	5.17
JW5	30.2	9.04	1028	0.49	1.018	1.641	0.09	0.64	0.19	8.20
JW6	28.5	8.91	1029	0.51	0.869	0.537	0.02	0.13	0.23	1.97
JW7	28.5	9.60	3102	1.32	2.085	0.527	0.02	0.14	0.11	6.13
JW8	28.7	8.87	1853	0.84	0.654	1.422	0.11	0.95	0.11	10.59
OW1	22.9	9.43	1125	0.64	0.306	3.140	0.21	1.00	0.36	9.18
OW2	13.1	8.92	1844	1.01	0.813	0.874	0.06	0.44	0.22	5.59
OW3	17.8	9.09	543	0.21	1.184	3.400	0.16	0.58	0.15	8.38
OW4	16.5	9.24	1078	0.70	1.392	0.570	0.04	0.47	0.33	4.38

OW5	18.1	8.76	973	0.50	0.769	2.370	0.14	0.95	0.42	7.19
OW6	25.4	8.72	841	0.49	0.677	0.609	0.03	0.27	0.78	2.31
OW7	19.1	9.49	1904	1.11	1.450	0.604	0.05	0.37	0.75	4.38
OW8	21.3	9.26	808	0.61	1.386	1.466	0.08	0.69	0.25	8.38

Note: AW, JW, and OW represent water samples collected in April, July, and October, respectively.

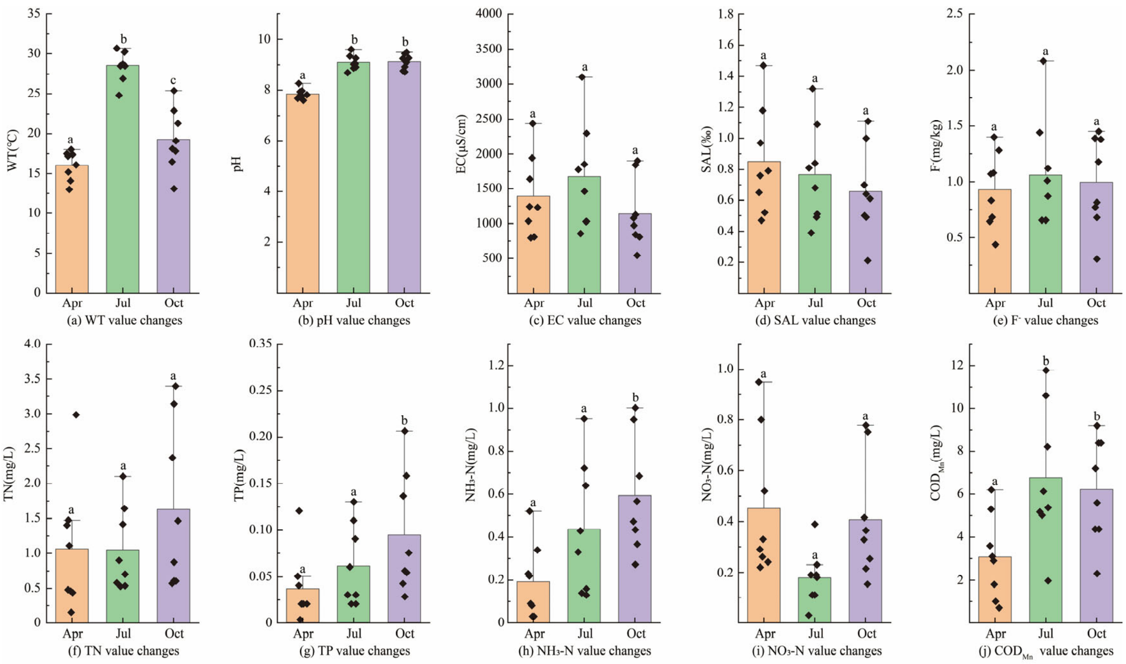

Figure S2. Changes in water physical and chemical indicators in different seasons.

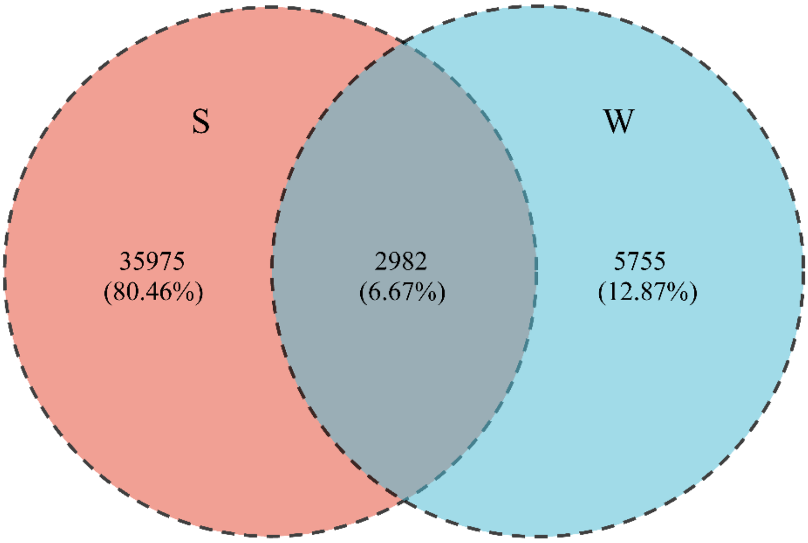

Figure S3. Number of bacterial ASVs in sediment (S) and overlying water (W) samples. The overlapping area represents shared ASVs between water and sediment samples.

https://doi.org/10.3390/xxxxx

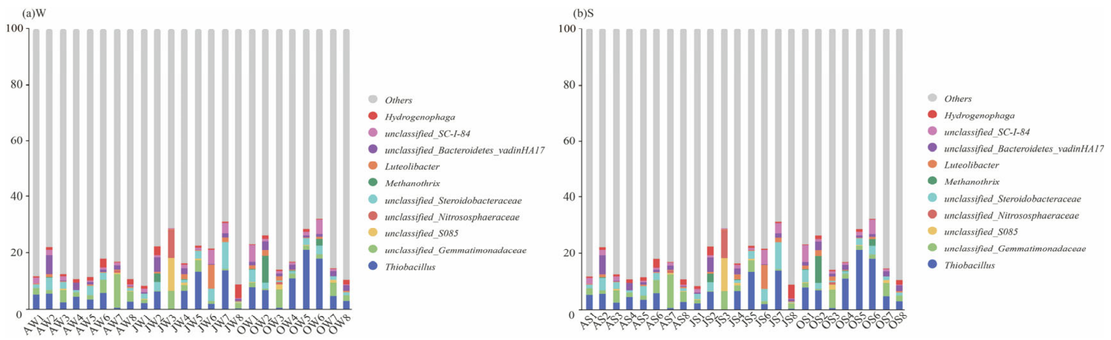

Figure S4. Genus-level composition of bacterial communities in water (a) and sediment (b) samples. Numbers 1–8 represent different sampling reservoirs. A, J, and O represent April, July, and October, respectively; W and S represent water and sediment samples, respectively.

Table S4. The results of PERMANOVA analysis among different seasonal groups.

group	df	R^2	F	*P*
AW vs JW	1	0.1139	1.8003	0.001
AW vs OW	1	0.0968	1.4999	0.013
JW vs OW	1	0.0897	1.3796	0.055
AS vs JS	1	0.0581	0.8640	0.877
AS vs OS	1	0.0948	1.4665	0.003
JS vs OS	1	0.0927	1.4304	0.003

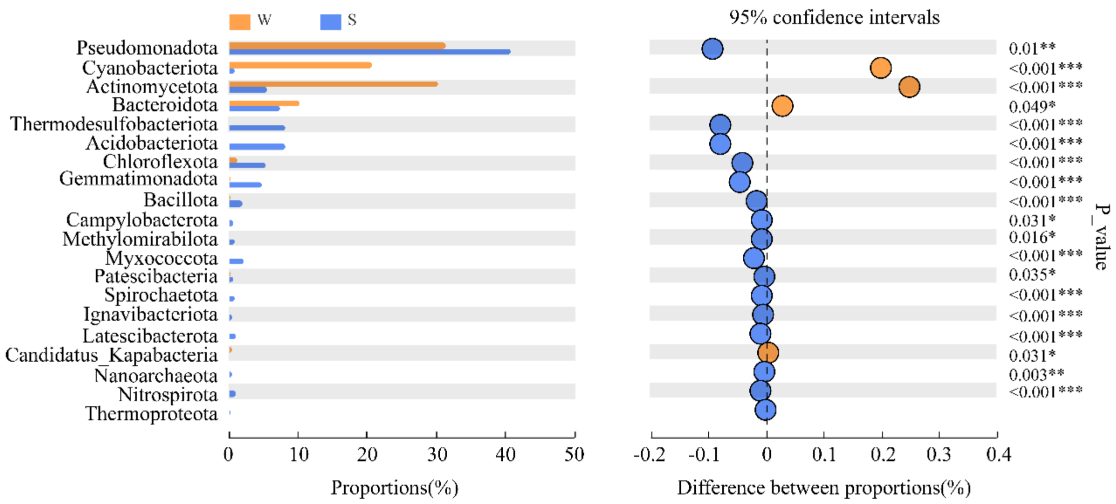

Figure S5. Differences in the relative abundance of dominant bacterial phyla between water and sediment samples. (n = 24 for water; n = 24 for sediment; n = 8 per habitat in each season; Significance levels are indicated as follows: *$P < 0.05$, **$P < 0.01$, and ***$P < 0.001$).

https://doi.org/10.3390/xxxxx
